# Gold nanoparticles loaded chitosan encapsulate 6-mercaptopurine as a novel nanocomposite for chemo-photothermal therapy on breast cancer

**DOI:** 10.1186/s13065-022-00892-0

**Published:** 2022-11-12

**Authors:** Amna H. Faid, Samia A. Shouman, Yehia A. Badr, Marwa Sharaky, Elham M. Mostafa, Mahmoud A. Sliem

**Affiliations:** 1 National Institute of Laser Enhanced Science (NILES), Cairo University, Giza, 12613 Egypt; 2 National Cancer Institute (NCI), Cairo, 11796 Egypt; 3 Chemistry Department, Faculty of Science and Arts, Taibah University, Al-Ula, 100823 Saudi Arabia

**Keywords:** Encapsulation efficiency, Chitosan loaded gold nanocomposites, 6-mercaptopurine, MCF7 cell line, Chemo-photothermal therapy

## Abstract

**Background:**

As a promising strategy to overcome the therapeutic disadvantages of 6-mercaptopurine (6MP), we proposed the encapsulation of 6MP in chitosan nanoparticles (CNPs) to form the 6MP-CNPs complexes. The encapsulation was followed by the loading of complexes on gold nanoparticles (AuNPs) to generate a novel 6MP-CNPs-AuNPs nanocomposite to facilitate the chemo-photothermal therapeutic effect.

**Methods:**

CNPs were produced based on the ionic gelation method of tripolyphosphate (TPP). Moreover, 6MP-CNPs composite were prepared by the modified ionic gelation method and then loaded on AuNPs which were synthesized according to the standard wet chemical method using trisodium citrate as a reducing and capping agent. The synthesized nanocomposites were characterized by UV–VIS spectroscopy, dynamic light scattering, Fourier transform infrared spectroscopy, and transmission electron microscopy. The potential cytotoxicity of the prepared nanocomposites on MCF7 cell line was carried out using Sulphorhodamine-B (SRB) assay.

**Results:**

Optimization of CNPs, 6MP-CNPs, and 6MP-CNPs-AuNPs revealed 130 ± 10, 200 ± 20, and 25 ± 5 nm particle size diameters with narrow size distributions and exhibited high stability with zeta potential 36.9 ± 4.11, 37, and 44.4 mV, respectively. The encapsulation efficiency of 6MP was found to be 57%. The cytotoxicity of 6MP-CNPs and 6MP-CNPs-AuNPs on breast cell line MCF7 was significantly increased and reached IC_50_ of 9.3 and 8.7 µM, respectively. The co-therapeutic effect of the nanocomposites resulted in an improvement of the therapeutic efficacy compared to the individual effect of chemo- and photothermal therapy. Irradiation of 6MP-CNPs and 6MP-CNPs-AuNPs with a diode laser (DPSS laser, 532 nm) was found to have more inhibition on cell viability with a decrease in IC_50_ to 5 and 4.4 µM, respectively.

**Conclusion:**

The Chemo-Photothermal co-therapy treatment with novel prepared nanocomposite exhibits maximum therapeutic efficacy and limits the dosage-related side effects of 6MP.

**Graphical Abstract:**

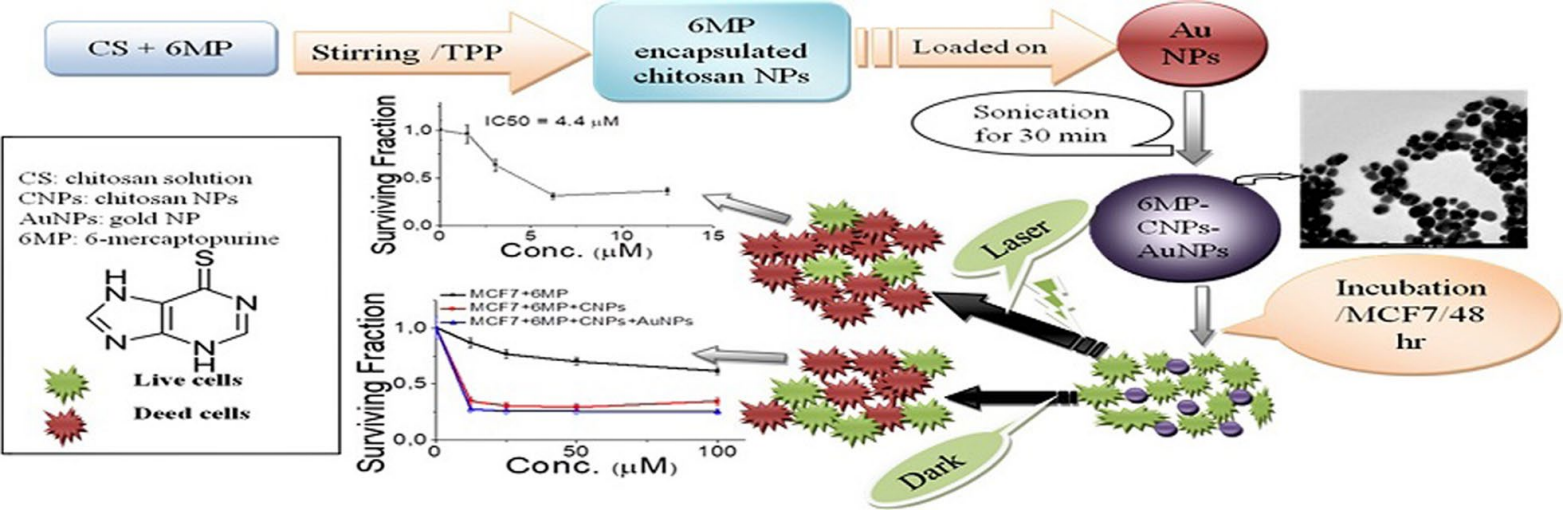

## Introduction

Cancer continues to be one of the most unsettling illnesses, endangering public health and leading to significant annual mortality rates across the globe [1]. Over the next 20 to 40 years, it is predicted that both cancer cases and fatalities would more than quadruple globally [2]. Chemotherapy is a common form of treatment. Chemotherapeutics’ primary therapeutic drawback, however, is their inability to discriminate between cancer and normal cells without visible harm to healthy tissues and cells. Therefore, it’s critical to create effective anticancer medications that target cancer cells alone while harming the fewest number of healthy cells [3]. Additionally, chemotherapy treatment results in multi-drug resistance (MDR), which may be related to cancer stem cells (CSCs). Chemotherapies employ a variety of non-specific and heterogeneous cytotoxic chemical agents [4].

Recently, there has been discussion of the attractiveness of using nanoparticles as drug delivery systems [5–7]. Nanoparticles have been created to deliver encapsulated anticancer drugs due to their unique desired qualities, such as their small size and high chemical reactivity, which led to high efficacy with few side effects [8]. The continuous and controlled release of therapeutic medications is made possible by nanoparticles acting as drug carriers, which helps to keep drug levels at the optimal level. They also confirm that the medications are localized and precisely targeted to their intended tissues and cells, resulting in a reduction in drug doses [9]. Chitin, the second most common natural polysaccharide after cellulose, is deacetylated to produce chitosan (CS) [10]. Due to its beneficial biological features and antibacterial and anticancer actions, it has recently gained a lot of interest in the pharmaceutical and biomedical domains [11]. The generation of chitosan nanoparticles (CNPs) relies on an ionic gelation approach in which nanoparticles are formed by electrostatic interactions between positively charged chitosan chains and polyanionic crosslinkers such as tripolyphosphate (TPP) [12]. CNPs are readily internalized into cells and more accurately deliver loaded or encapsulated drugs into cells. This increases the specificity of CNP. This is why CNP has widespread therapeutic importance [13–16]. Encapsulation of active ingredients in polymer matrices protects them from the surrounding medium and helps control their release [17, 18]. The main advantages of using chitosan for drug delivery are: toxicity is less, enhanced biocompatibility, possess mucoadhesive character, possess stability, drug targeting is site-specific, and therapeutic index of the drug is increased even though chitosan exhibit some disadvantages compared to other natural materials as mechanical resistance is less, low solubility in neutral and alkaline pH and difficulty in controlling pore size [19, 20].

Photothermal therapy (PTT) is an applied therapeutic approach where light irradiation is converted to heat by photothermal agents, thus increasing the temperature of particular tissues [21]. PTT selectively destroys cancer cells which are more sensitive to an increase in temperature [22]. Recently, nanoscale-based photothermal agents, such as gold nanoparticles (AuNPs), with higher absorption and photostability have been used in PTT. In PTT, the interaction between an electromagnetic wave and free electrons at the AuNPs surface causes them to oscillate coherently in resonance with the frequency of visible light. This phenomenon greatly enhances both the scattering and the absorption of light by the AuNPs suitable for different biomedical applications [23, 24]. Using a combination therapy approach based on the synergistic interaction between heat in PTT and cytotoxic therapy with anticancer drugs has been shown to have clear advantages [25–27]. AuNPs are excellent tools in cancer diagnosis and therapy due to their high biocompatibility, minimal toxicity, excellent penetration into cancer tissues, and most importantly, non-immunogenicity in the human body. In addition, it has unique physical and chemical properties that give it special advantages such as: B. Nanoscale, surface, quantum, electrical and optical effects. Despite their many advantages, AuNPs have several limitations that limit their pharmaceutical applications, including: the toxicity of bare AuNPs depends on numerous parameters such as composition, shape, size, coating, charge, hydrophobicity, solubility, reactivity, and evolution of biological media properties. Studies have shown that bare AuNPs tend to aggregate inside lysosomes, which alters their optical properties and alters their activation under radiation. Therefore, additional organic or biological surface coatings are required to stabilize AuNPs [28, 29].

6-Mercaptopurine (6MP) is a purine antilog with antitumor and immunosuppressive properties [30]. 6MP is widely used to treat acute lymphoblastic leukemia (ALL) and inflammatory diseases [31]. 6MP is metabolized intracellularly to methylated thioinosinic acid, which inhibits de novopurine synthesis, and is subsequently converted to the DNA intercalator 6-thioguanine [32]. Like other chemotherapeutic agents, 6MP has various side effects such as allergic reactions and hepatotoxicity [33]. Minimal anti-tumor effects have been observed with 6MP in the treatment of cancerous solid tumors of breast cancer [34–36]. Therefore, there is interest in developing more frequent drug delivery systems to overcome these critical problems [37]. In recent years, researchers have made several attempts to overcome these limitations. Among these attempts, nanotechnology-based approaches have been particularly helpful in mitigating the problem. Presently reported nanocarriers of 6-MP mainly chemically link 6-MP or 6-MP derivatives with polymers including chitosan, carboxymethyl chitosan (CMCS) and dendrimers, which inevitably use crosslinkers. Prepared by binding. In addition, experts have used magnetic materials such as metal vectors, mesoporous silica, and iron oxide to produce 6 MP nanoparticles. Some researchers have modified these nanoparticles with hyaluronic acid and folic acid to enhance the ability of nanomedicine to target tumors and cells [38].

The aim of the present study is to synthesize and characterize chitosan-encapsulated 6MP with subsequent loading on AuNPs to generate a novel ternary system of 6MP-CNPs-AuNPs nanocomposites for the first time. Anticancer activities of the proposed nanocomposites in the human breast cancer cell line (MCF-7) were studied. The possibility of combining chemo- and photothermal therapy with reducing the side effects and increasing the therapeutic efficiency of 6MP was presented as well.

## Materials and methods

### Chemicals

All the glassware was washed with aqua regia [HCl:HNO_3_ = 3:1 (v/v)] and then rinsed with deionized water. Tetrachlorauric acid (HAuCl_4_·3H_2_O), 6-mercaptopurine, chitosan low molecular weight (mol wt 50,000–190,000 Da), dimethylsulphoxide (DMSO), RPMI-1640 medium, sodium bicarbonate, trypan blue, fetal Bovine Serum (FBS), trypsin, acetic acid, sulphorhodamine-B (SRB), trichloroacetic acid (TCA) and tris base 10 mM (PH 10.5) were obtained from Sigma Aldrich Chemical Co., St. Louis, Mo, USA.

The human breast carcinoma cell line (MCF7) was obtained from the American Type Culture Collection (ATCC, MO, USA). The tumor cell line was propagated and maintained by serial sub-culturing in RPMI-1640 medium containing 10% FBS and 1% penicillin/streptomycin.

### Preparation of chitosan nanoparticles CNPs

CNPs were produced based on the ionic gelation method of tripolyphosphate (TPP) with chitosan (CS) according to the methodology previously developed [39]. Chitosan was dissolved in 0.6% (w/v) with 1% (v/v) acetic acid solution. The pH of the chitosan solution was raised to 4.6–4.8 with 1 N NaOH. Chitosan nanoparticles formed spontaneously upon the addition of an aqueous TPP solution (0.3% w/v) to the prepared chitosan solutions under magnetic stirring at 800 rpm for 30 min at room temperature. To optimize the method of CNPs preparation, the different volumetric ratios between chitosan: TPP (1:1, 2:1, 3:1, 4:1) were applied. All the prepared chitosan nanoparticles were investigated in terms of particle size and zeta potential.

### Preparation of chitosan-encapsulated 6MP (6MP-CNPs complexes)

6MP-CNPscomplexes were prepared by the modified ionic gelation method [40]. Low molecular weight chitosan was dissolved in 1% (v/v) acetic acid with 0.6% (w/v) ratio. 5 ml of the prepared chitosan solution were mixed with 1 ml of 10^–4^ M 6MP (6MP dissolved in water). PH was raised to 4.6–4.8 with 1 N NaOH. The prepared mixed solution of 6MP and chitosan was mixed with 2.5 ml of 0.3% (w/v) TPP solution with a volume ratio of (2:1) (v/v) (chitosan: TPP) under gentle stirring at 800 rpm for 30 min at room temperature.

### Evaluation of 6MP encapsulation efficiency

The encapsulation efficiency of 6MP-CNPs was determined by the indirect method. Briefly, the solution of 6MP encapsulated CNPs was centrifuged at 5000 rpm for 1 h and then the supernatant was measured by UV–VIS spectrophotometer at 340 nm. Absorption of the parent concentration of 6MP was measured as well. Calculations were performed by using the calibration curve, and encapsulation efficiency were was calculated as follows:

$$ 6{\text{MP}}\;{\text{encapsulation}}\;{\text{efficiency}}\;( \%) = \frac{{{\text{total}}\;6{\text{MP}} - {\text{free}}\;6{\text{MP}}}}{{{\text{total}}\;6{\text{MP}}}} \times 100. $$

### Preparation of AuNPs by citrate reduction

The AuNPs were synthesized successfully according to the standard wet chemical method [41] using trisodium citrate as a reducing agent; 20 ml of 1 mM HAuCl_4_ solution was heated to boil and refluxed while being stirred. 2 ml of a 38.8 mM trisodium citrate solution (0.1141 g trisodium citrate in 10 ml water) was added quickly. The solution color changed from yellow to deep red. When the color of the solution changed, the heater was turned off and the solution was left to stir until reaching room temperature.

### Preparation of 6MP-CNPs-AuNPs nanocomposites

3 ml of the as-prepared 6MP-CNPs complexes were mixed with the assistance of sonication with 1 ml of the as-prepared AuNPs. The color was changed from ruby red to reddish blue. Particle size and shape of the prepared nanocomposites were investigated. Additionally, FTIR was utilized to collect information about the encapsulation and loading processes.

## Characterization techniques

### UV–Visible spectroscopic analysis

Absorption spectra of the synthesized AuNPs and 6MP-CNPs-AuNPs nanocomposites were monitored using double beam spectrophotometer *(PG instrument, T80*^+^*, UK)*. 200 µl from the prepared solutions were diluted to 2 ml with distilled water, then transferred to 1 cm UV-quartz cell and the absorption spectra were recorded within the appropriate scan range (200 nm to 800 nm).

### TEM measurements

TEM images of the prepared nanomaterials were captured on Tecnai G20 transmission electron microscope (TEM; FEI, Netherland) coupled with CCD camera model AMT operating at 200 kV. Drops from a very dilute solution were deposited on an amorphous carbon-coated copper grid and left to evaporate at room temperature forming a monolayer, then detected by TEM.

### Dynamic light scattering (DLS) analysis

The particle size and surface charges of CNPs, AuNPs, 6MP-CNPs, and 6MP-CNPs-AuNPs nanocomposites were analyzed through DLS with Zeta sizer 300 HAS (Malvern Instruments, Malvern, UK) based on photon correlation spectroscopy. Analysis time was 60 s and the average zeta potential was determined. The zeta potential of nanoparticulate dispersion was determined as such without dilution.

### FTIR analysis

FTIR measurements were carried out using FT-IR spectrometer (4100 Jasco-Japan) in the range of 500–4500 cm^−1^. The prepared samples 6MP, CNPs, 6MP-CNPs, AuNPs, and 6MP-CNPs-AuNPs were freeze dried using lyophilizer. IR spectra of powdered samples diluted with potassium bromide (KBr) were measured.

### The potential cytotoxicity on MCF7 cell line

This method was carried out according to Skehan et al.’s study [42] using Sulphorhodamine-B (SRB) assay. Cells were seeded in 96-well microtiter plates, at a concentration of 5 × 10^3^ cell/well in a fresh medium, and left to attach to the plates for 24 h. Cells were incubated with the same concentrations of free 6MP, 6MP-CNPs, and 6MP-CNPs-AuNPs (12.5, 25, 50, and 100 µM) for 48 h. The cells were fixed with cold 50% trichloroacetic acid for 1 h at 4 °C and washed with distilled water and stained with 0.4% SRB. The wells were washed with 1% acetic acid, air-dried and the dye was solubilized with 10 mM tris base (pH 10.5) using a shaker. The optical density (O.D.) of each well was measured spectrophotometrically at 570 nm with an ELIZA microplate reader (Meter tech. Σ 960, USA). The mean background absorbance was automatically subtracted and mean values of each drug concentration were calculated. The percentage of cell survival was calculated as follows: Survival fraction = O.D. (treated cells)/O.D. (control cells). The IC_50_ values (the concentrations that produced 50% inhibition of cell growth) were calculated. The experiment was repeated 3 times independently and each concentration was repeated three times.

### Photothermal/chemotherapy combined treatment

For laser irradiation, a second harmonic Nd: YAG DPSS laser with a wavelength of 532 nm and power of 100 mW was used. Cells were seeded as previously described. The cells were treated with different concentrations (1.5, 3.1, 6.2, and 12.5 µM) of 6MP-CNPs and 6MP-CNPs-AuNPs and left for 48 h, then exposed to laser for 6 min. At the end of exposure time, cells were fixed and tested for cytotoxicity as previously described.

### Statistical analysis

Graphs and data analysis were performed using Graphpad InStat, Version 5. The results are expressed as mean ± SD of 3 separate experiment performed in 5 replicates. The statistical significance of the results was analyzed using one-way ANOVA followed by Tukey multiple comparison test. ^a^Significantly different from its untreated control at p < 0.05.

## Results

### Characterization of the as-prepared CNPs and 6MP-CNPs complexes

#### Size, zeta potential, morphological analysis, and encapsulation efficiency

**Fig. 1:**
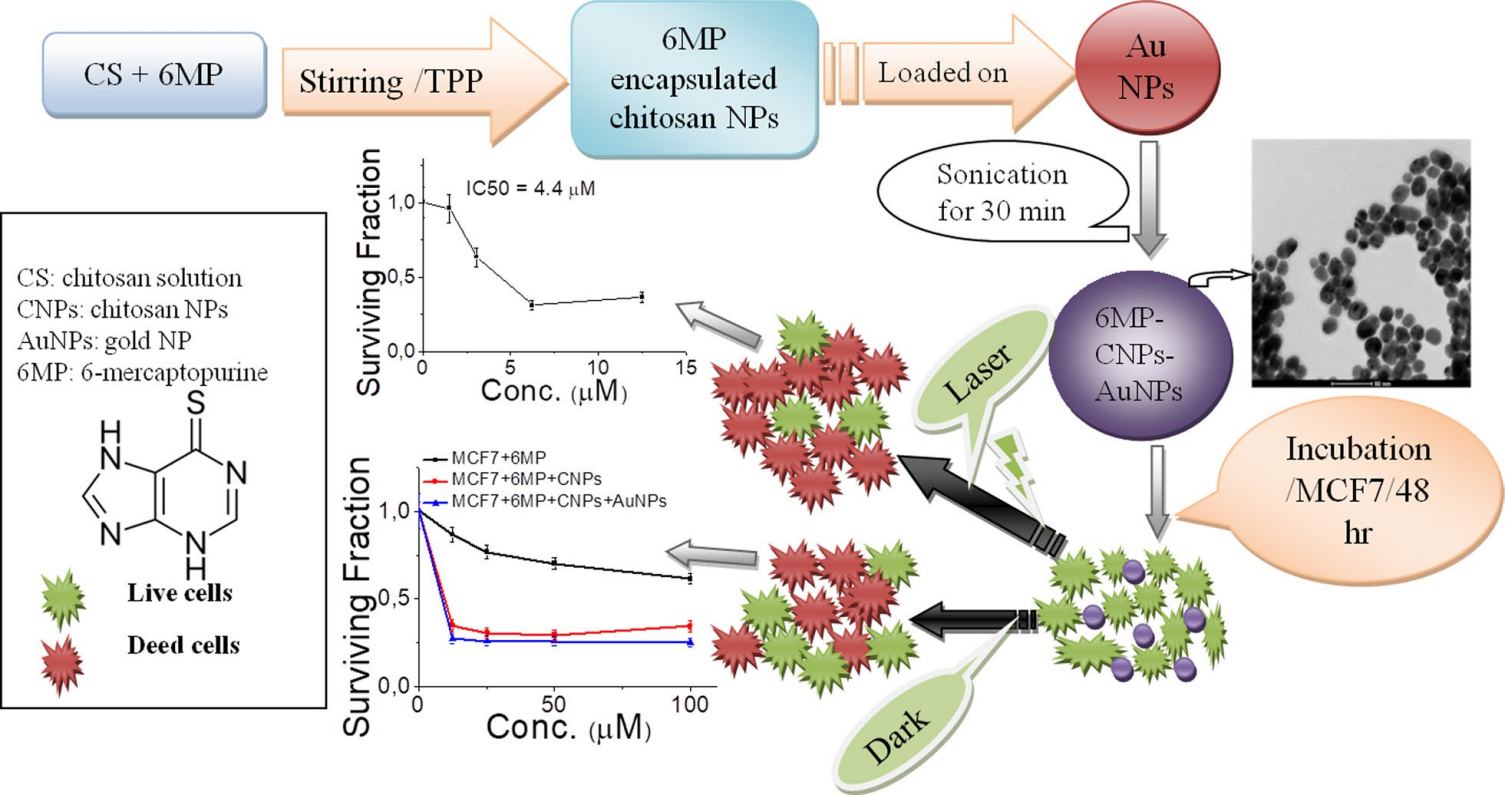
Illustration of preparation of chitosan-encapsulated 6MP with subsequent loading on AuNPs to form 6MP-CNPs-AuNPs nanocomposites. As well as treatment of MCF7 with the as-prepared nanocomposites in absence and presence of DPSS laser for chemo-photothermal therapy

Zeta potential and particle size are two important characteristics of nanoparticles. Size distribution and stabilization of particles were determined by zetasizer. CNPs were prepared based on an ionic gelation interaction between positively charged CS and negatively charged TPP (Fig. 1). Important properties of nanoparticles such as particle size or surface charge can be easily manipulated by changing parameters such as concentration of chitosan, chitosan-to-polyanion concentration ratio, and solution pH [43]. The average size of the hydrodynamic diameter and zeta potential of CNPs suspension were analyzed using Zetasizer analysis. Table 1 illustrates the effect of CS and TPP concentrations on particle size and zeta potential of the prepared CNPs. The mean particle size of CNPs ranged from 19 to 140 nm with a relatively narrow particle size distribution with PDI values ranging from 0.3 to 0.4. The value of zeta potential can also determine the nanoparticle interaction in vivo with the cell membrane of the cancer cell which is usually negatively charged. Table 1 shows that the surfaces of chitosan nanoparticles have a positive charge ranging from 10.7 to 36.9 mV with different volumetric ratios between chitosan and TPP.

**Table 1:** Average particle size (nm), zeta potential (mV) and polydispersity index (PDI) of CNPs with different ratios of CS and TPP

Chitosan: TPP ratio (v/v)	Average particle size (nm)	Polydispersity index (PDI)	Zeta potential (mV) (mean ± SD)
1:1	37	0.356	16.2 ± 4.08
2:1	140	0.443	36.9 ± 4.11
3:1	65.70	0.440	24.3 ± 4.50
4:1	19.31	0.450	10.7 ± 5.21

**Fig. 2:**
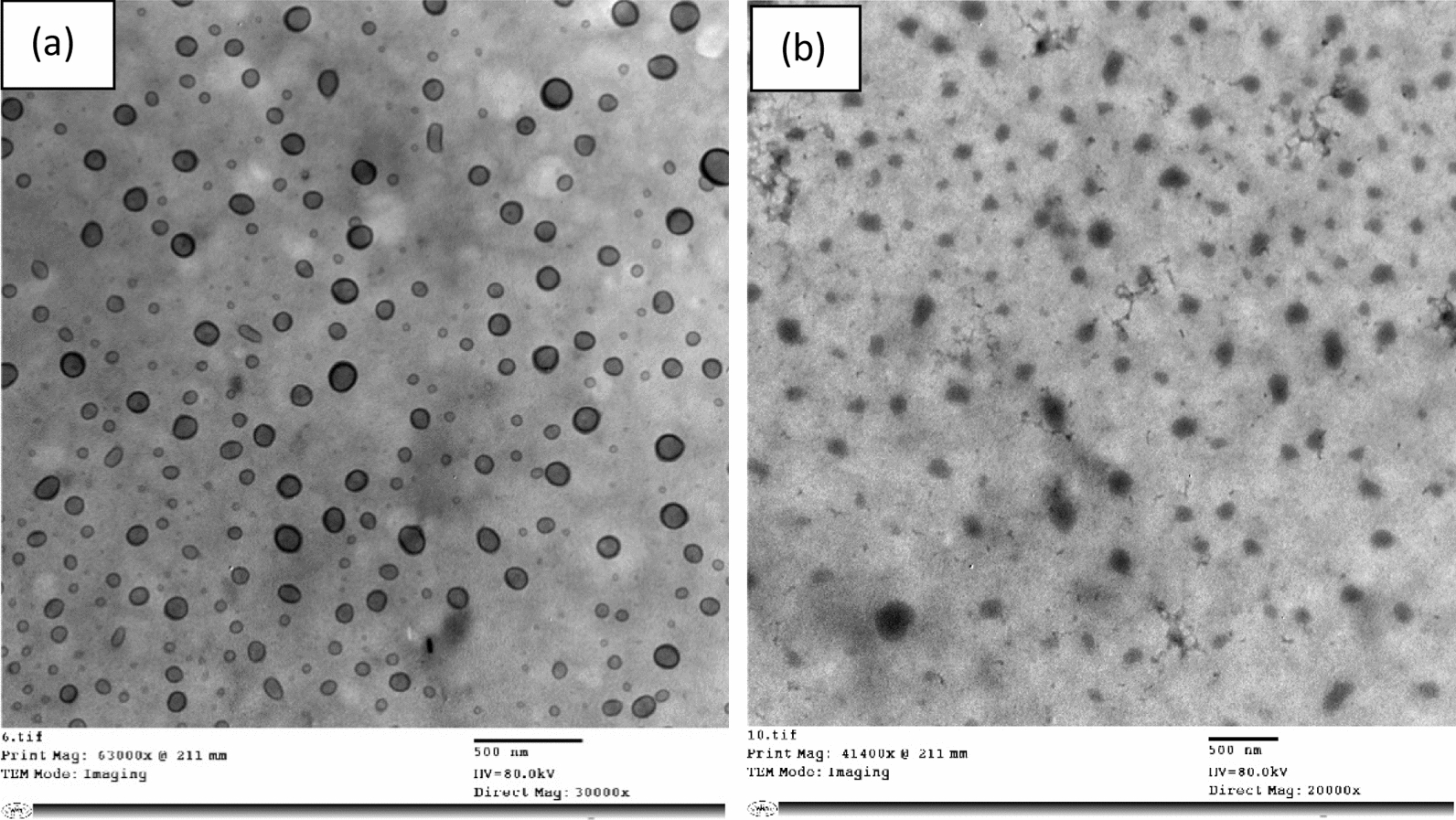
TEM images of **a** chitosan nanoparticles with ratio 2:1 and **b** 6MP encapsulated chitosan NPs. (Scale bar = 500 nm)

Figure 2 shows TEM images of CNPs and 6MP-CNPS complexes. CNPs (Fig. 2a) have nearly spherical shapes with particle sizes of about 130 ± 10 nm. TEM image of 6MP-CNPs (Fig. 2b) reveals a slight change in particle size with an average particle diameter of about 200 ± 20 nm [44, 45]. Encapsulation efficiency is defined as the percentage of 6MP encapsulated content that can be entrapped into CS/TPP nanoparticles. The encapsulation efficiency of 6MP encapsulated chitosan nanoparticles was found to be 57%.

#### FTIR analysis

**Fig. 3:**
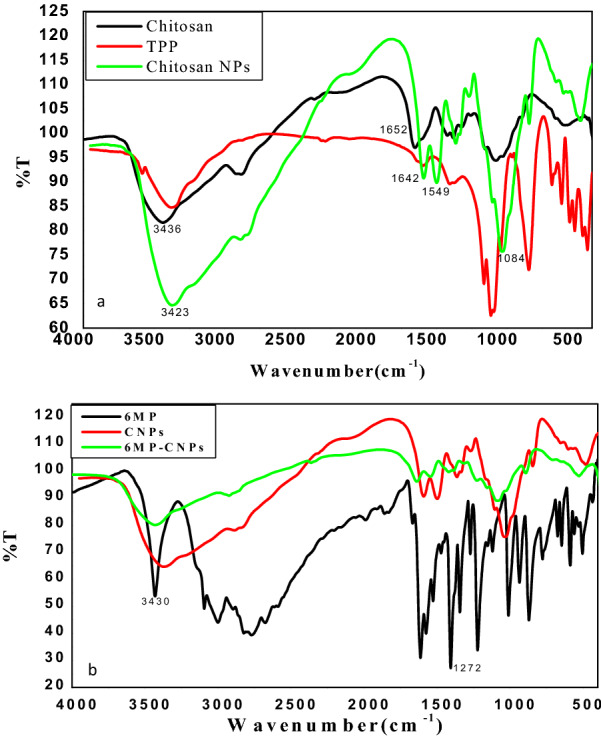
FTIR spectra of **a** CS, TPP and CNPs, **b** 6MP, CNPs and 6MP-CNPs

The prepared CNPs were further confirmed by FTIR spectroscopic measurements. FTIR studies of CS, TPP, and CNPs were performed to investigate the chemical structure of the prepared nanoparticles. As shown in Fig. 3a, there were many characteristic peaks for CS; the peak at 3436 cm^−1^ was attributed to hydrogen-bonded O–H, and the peaks of primary amine and type II amide were overlapped in the same spectral range. The peak at 1081 cm^−1^ corresponded to *ν*(C–O–C) and that at 1652 cm^−1^ was attributed to the CONH_2_ group [46]. In the FTIR spectrum of TPP, many characteristic bands were observed; 1214 cm^−1^ (P=O stretching), 1148 cm^−1^ (symmetric and antisymmetric stretching vibrations in PO_2_ group), 1093 cm^−1^ (symmetric and antisymmetric stretching vibrations in PO_3_ group), 912 cm^−1^ (antisymmetric stretching of the P–O–P bridge). The FTIR spectrum of CNPs was different from that of chitosan polymer (CS). In CNPs, the peak at 3436 cm^−1^ in CS was shifted to 3423 cm^−1^ and became wider with a relative increase in intensity. The peak at 1652 cm^−1^ in CS was shifted to 1642 cm^−1^ with a decrease in intensity. CNPs also showed a P=O peak at 1154 cm^−1^.

To confirm the encapsulation of 6MP in CNPs, FTIR measurements were done on 6MP, CNPs, and 6MP-CNPs. As shown in Fig. 3b, CNPs have main characteristic absorption bands at 1549 cm^−1^ and 1414 cm^−1^ which were assigned to N–H bending vibration and C–O stretching of the alcohol group, respectively [47]. The bands at 1084 cm^−1^, 2937 cm^−1^, and 3423 cm^−1^ were assigned to C–N, C–H, and N–H stretching vibrations, respectively.

Furthermore, the peak observed for 6MP at 3430 cm^−1^ which was assigned to the N–H stretching was shifted to 3435 cm^−1^ in the 6-MP-CNPs complexes. The peak at 1120 cm^−1^, which corresponds to the vibration of C=S/ring in 6MP, disappeared after the encapsulation process. The band at 1272 cm^−1^ of 6MP, which was assigned to C=S, was missed after encapsulation.

### Characterization of 6MP-CNPs-AuNPs nanocomposite

#### Size, zeta potential, and morphological analysis

**Fig. 4:**
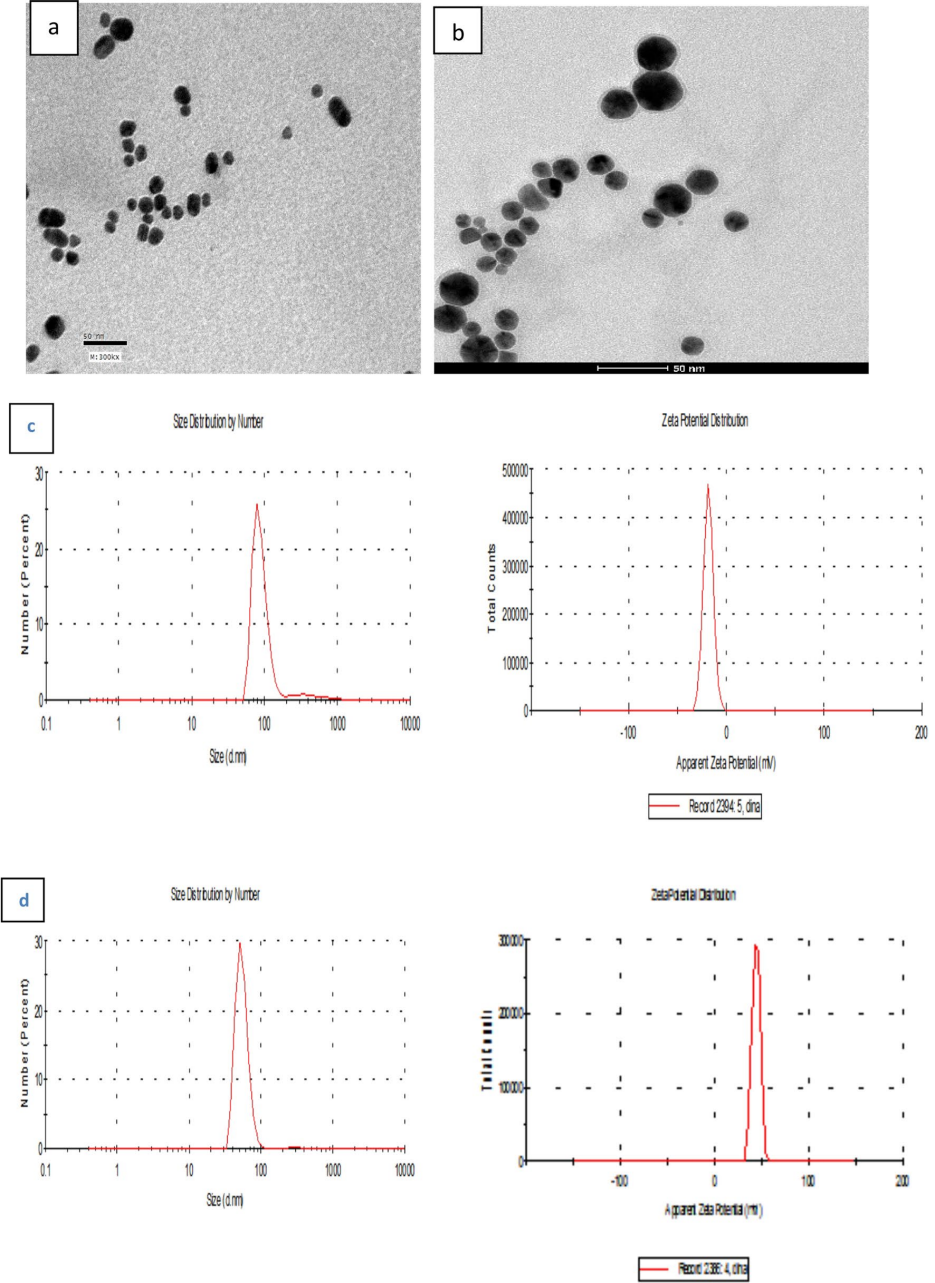
TEM images of **a** AuNPs prepared by citrate reduction left, and **b** 6MP-CNPs-AuNPs nanocomposites right. **c** Size distribution and zeta potential of AuNPs reduced by citrate. **d** Size distribution and zeta potential of 6MP-CNPs loaded AuNPs

The mean particle size and zeta potential of AuNPs were measured to be 87.5 nm and − 18.5 mV, respectively. The as-prepared 6MP-CNPs-AuNPs nanocomposite showed high stability with a zeta potential of 44.4 mV as shown in Fig. 4c, d. TEM micrograph of Fig. 4a showed the presence of spherical AuNPs of approximate size 12 ± 2 nm with uniform size distribution. Figure 4b shows the TEM image of the ternary nanocomposite (6MP-CNPs-AuNPs). The formed nanocomposites possessed a regularly spherical shape and smooth surface with an increase in the particle size from 12 ± 2 to 25 ± 5 nm. Upon combination of 6MP-CNPs and AuNPs, particle size was increased as estimated from TEM measurements.

#### FTIR analysis

**Fig. 5:**
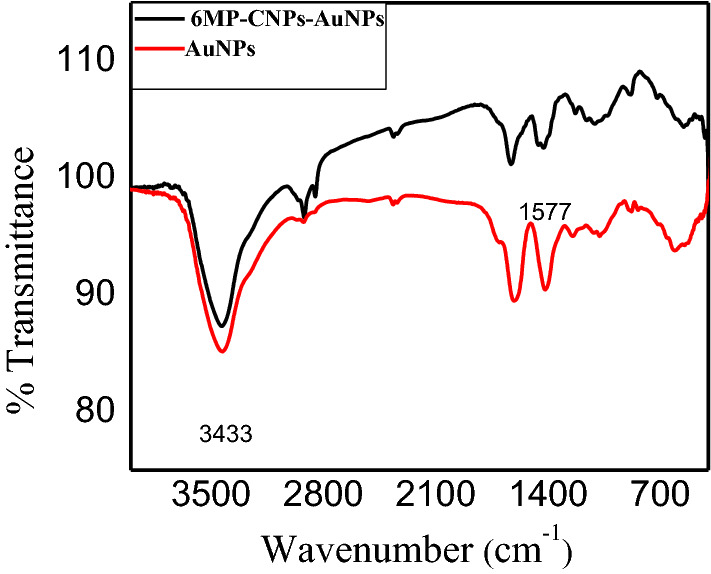
FTIR spectra of AuNPs, 6MP-CNPs complexes and 6MP-CNPs-AuNPs nanocomposites

The loading of 6MP-CNPs on AuNPs was further investigated using FTIR studies. Figure 5 shows FTIR spectra of AuNPs, 6MP-CNPs, and 6MP-CNPs-AuNPs nanocomposites. AuNPs prepared by citrate reduction depicted characteristic bands of citrate at 3429.78, 1244.83, 609.39, and 1078 cm^−1^ which were assigned to the stretching vibration of OH group, C–O stretching, C=O stretching, and CO–O–C symmetric stretching, respectively [48]. After loading 6MP-CNPs on AuNPs, there was a decrease in intensity of all bands, and combined peaks of the NH_2_ and OH group stretching vibration were broadened with a slight shift to lower wavelengths at 3433 cm^−1^.

#### UV–visible spectral analysis

**Fig. 6:**
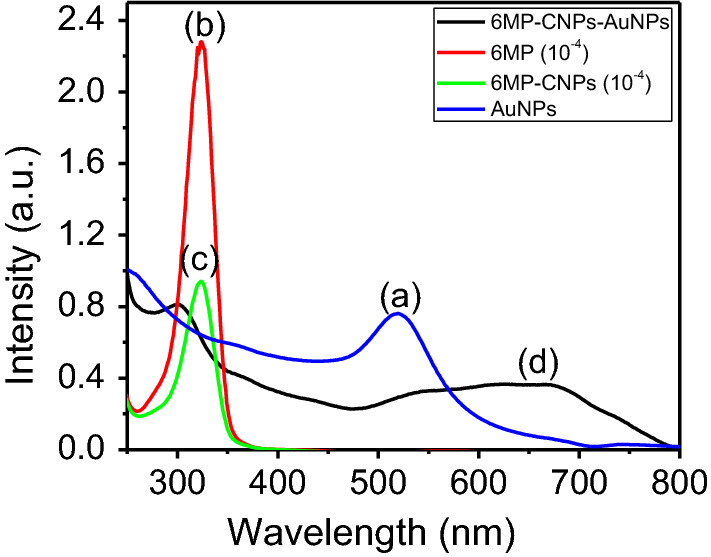
UV–VIS absorption spectra of **a** AuNPs, **b** 10^4−^ M 6MP, **c** 10^4−^ M 6MP encapsulated CNPs and **d** 6MP-CNPs loaded AuNPs with volumetric ratio 3:1

The encapsulation of 6MP in CNPs, as well as the loading on AuNPs, was investigated via UV–visible spectroscopy as shown in Fig. 6. The band in the UV region that appeared at 321 nm was raised from the n–π* transition of the 6-MP molecule [49]. This band was decreased in intensity for 6MP-CNPs. Upon loading of 6MP-CNPs on the surface of AuNPs, the absorption spectrum of AuNPs, at about 520 nm, showed redshift with broadening and decrease in absorbance intensity. The appearance of a new band at around 680 nm is probably assigned to the plasmon coupling between two or more adjacent AuNPs [50].

### In vitro cytotoxicity assay

#### Effect of different concentrations of 6MP, 6MP-CNPs complexes, and 6MP-CNPs-AuNPs nanocomposites on cellular proliferation of MCF7 cell line

**Fig. 7:**
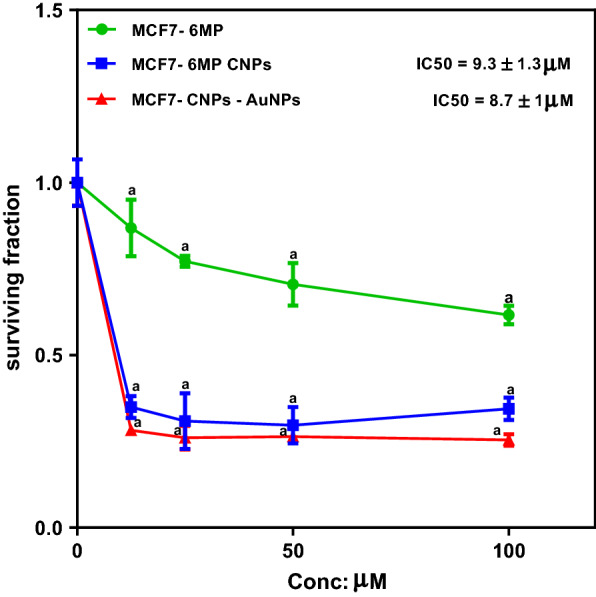
Cytotoxicity of different concentrations of 6MP, 6MP-CNPs complexes and 6MP-CNPs-AuNPs nanocomposites on MCF7 cell line after 48 h

To examine the antitumor activity on MCF7, exponentially dividing cells were treated with increasing concentrations (12.5, 25, 50, and 100 µM) of native 6MP, 6MP-CNPs, and 6MP-CNPs-AuNPs. The cell viability was measured as shown in Fig. 7. There was a concentration-dependent decrease in cellular proliferation compared to its respective control. It was observed that 6MP produced a decrease in cell survival and reached a maximum value of about 39% at 100 µM. A significant decline in cell viability was demonstrated in the case of 6MP-CNPs and 6MP-CNPs-AuNPs versus the native 6MP. As can be shown from Fig. 7, 6MP-CNPs-AuNPs ternary nanocomposite produced more inhibition where the IC50 value decreased to 8.7 μM as compared to 9.3 μM in the case of 6MP-CNPs.

#### Chemo-photothermal combined treatment

**Fig. 8:**
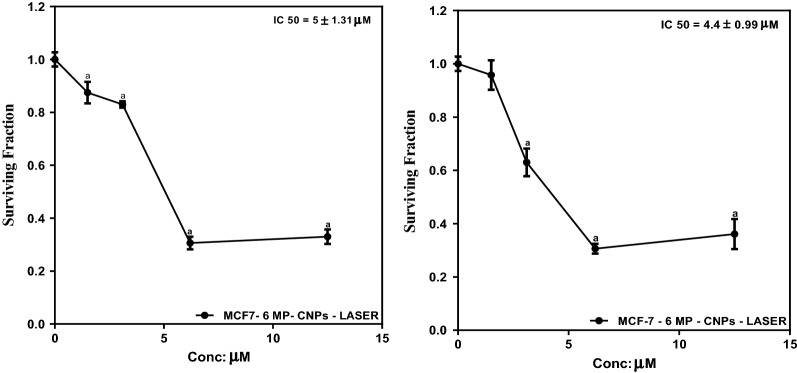
The effect of DPSS laser irradiation (532 nm, 200 mW) on MCF7 by sulphorhodamine-B assay incubated with different concentrations (1.5, 3.1, 6.2 and 12.5 µM) of **a** 6MP-CNPs and **b** 6MP-CNPs-AuNPs for 6 min. Graphs and data analysis were performed using Graphpad InStat, Version 5. The results are expressed as mean ± SD of 3 separate experiment performed in 5 replicates. The statistical significance of the results was analyzed using one-way ANOVA followed by Tukey multiple comparison test. ^a^Significantly different from its untreated control at p < 0.05

The effect of DPSS laser exposure on MCF7 survival percentage after 48 h incubation with different concentrations (1.5, 3.1, 6.2, and 12.5 µM) of 6MP-CNPs and 6MP-CNPs-AuNPs was revealed in Fig. 8. After laser irradiation, measurements showed a decline in cell viability with a subsequent decrease in IC_50_ to 5 and 4.4 µM for 6MP-CNPs and 6MP-CNPs-AuNPs, respectively compared to IC_50_ of 9.3 and 8.7 µM in absence of laser irradiation. There was a significant decrease in IC_50_ between 6MP-CNP and 6MP-CNP treated with laser and between 6MP-CNPs-AuNPs and 6MP-CNPs-AuNPs treated with laser. While there wasn’t a significant decrease in IC_50_ between 6MP-CNP and 6MP-CNPs-AuNPs without laser and 6MP-CNP and 6MP-CNPs-AuNPs treated with laser.

## Discussion

### Characterization of the as-prepared CNPs and 6MP-CNPs complexes

#### Size, zeta potential, morphological analysis, and encapsulation efficiency

The formation of CNPs occurs spontaneously through the formation of intra- and intermolecular cross-linkages under a constant stirring at ambient temperature [51]. TPP is a multivalent anion that possesses negative charges, and on the other hand, chitosan in acidic solution has amino groups that can undergo protonation [52]. During the preparation process, TPP is electro-statically attracted to the NH_3_^+^ groups in CS to produce ionically cross-linked CNPs.

Zeta potential (ZP) which is related to surface charge can greatly influence particle stability in suspension through the electrostatic repulsion between the prepared particles. In other words, the physical stability of the particles is highly affected by the value of zeta potential. A minimum ZP value required for good physical stability should exceed 30 mV to have NPs with strong cationic character [53]. Data cited in Table 1 reveals that ratio 2:1 CS: TPP has the highest zeta potential of about 36.9 ± 4.11 mV with a small particle size of about 140 nm and acceptable PDI of 0.44. The charge density of nanoparticles plays an important role in its binding with negatively charged cancer. Positively charged nanoparticles are preferred for the preparation of drug delivery systems in the therapy of cancer cell membrane [54]. Commonly, particle aggregation is less likely to occur for charged particles with optimum zeta potential ≥ 30 mV due to electrostatic repulsions [55]. Consequently, ratio 2:1 was selected for encapsulation of 6MP drug in CNPs. 6MP encapsulated CNPs were prepared by a modified ionic cross-linking method between TPP and CS solutions. The as-prepared 6MP encapsulated CNPs (6MP-CNPs complexes) show high stability with zeta potential + 37 mV.

The size range of CNPs with a size range of about 130 ± 10 nm (Fig. 2a) confirms the respective average diameters measured by Zetasizer (140 nm). The PDI value of chitosan nanoparticles was 0.44, thus indicating a narrow and favourable particle size distribution (Table 1). The particle size of 6MP-CNPs was found to be 200 ± 20 nm. The change in the size of 6MP-CNPs is probably due to the interaction and encapsulation of 6MP within CNPs, which induce the aggregation of CNPs.

#### FTIR analysis

In CNPs, the shift in the peak at 3436 cm^−1^ in CS to 3423 cm^−1^ with a relative increase in intensity indicated the enhancement of the hydrogen bonding. The appearance of a new sharp peak at 1549 cm^−1^, which corresponds to N–H (Bend), indicated the involvement of –NH_2_ groups of chitosan in the particulation process to form CNPs. The strong and sharp peak of phosphate at 1084 cm^−1^ in CNPs confirmed the involvement of TPP during nanoparticle creation. These results can be attributed to the linkage between the phosphoric group of TPP and the protonated amine group of chitosan in CNPs [56]. The disappearance of the peak at 1120 cm^−1^, corresponding to vibration of C=S/ring in 6MP, after the encapsulation process clearly suggests the participation of an exocyclic (S) atom with CNPs to expose adsorption and/or encapsulation process of 6MP with CNPs [57, 58]. The missing of band at 1272 cm^−1^ of 6MP confirms the existence of a complex form through the sulphur atom. These changes are probably due to the existing adsorption and/or encapsulation process of 6-MP and CNPs.

### Characterization of 6MP-CNPs-AuNPs nanocomposite

#### Size, zeta potential, and morphological analysis

The negative value of the zeta potential of AuNPs was due to the presence of three deprotonated anionic carboxyl groups of citrate ions which verify the repulsive interaction between nanoparticles and aim to prevent the agglomeration of the prepared NPs. For AuNPs, it is noted that the size estimated from TEM microscopy is smaller than that obtained from the dynamic light scattering method. This can be explained in terms of the lack of the hydration shell during the TEM particle size determination [59]. The ternary nanocomposite (6MP-CNPs-AuNPs) is formed either by the formation of the complete shell of 6MP-CNPs on the surface of AuNPs or by coupling the particles in a side-by-side regime. Here, both phenomena of complete shell and plasmonic coupling appeared clearly in Fig. 4b. The size of the formed ternary nanocomposites (25 ± 5 nm) was found smaller than that of the corresponding 6MP-CNPs nanocomplexes (200 ± 20 nm) after combination. This can be explained in terms of reconfiguration of the size of 6MP-CNPs nanocomplexes in presence of AuNPs under the action of an ultrasound bath which induces the decrease of the size of CNPs [60].

#### FTIR analysis

It is well known that gold has a strong affinity toward amino groups [61]. Amide I band in 6MP-CNPs at 1577 cm^−1^ disappeared. The disappearance of the amide I band in 6MP-CNPs at 1577 cm^−1^ after loading confirms the involvement of the NH group in the binding of 6MP-CNPs on the AuNPs surface.

#### UV–visible spectral analysis

The citrate stabilized AuNPs were prepared and the presence of citrate layers provided enough electrostatic repulsion between individual particles to keep them well dispersed in the medium and prevent further growth of the particles [62]. The combination of the unique surface plasmon resonance property of AuNPs and their strong affinity toward NH groups serves as a good strategy to obtain the nanocomposite. The decrease in the intensity of the UV band of 6-MP in 6MP-CNPs is due to the encapsulation of 6MP in CNPs. Upon loading of 6MP-CNPs on the surface of AuNPs, the shift in the plasmon band of AuNPs was due to the change in the dielectric constant in the adsorption layer, the decrease in the interparticle distance to less than approximately the average particle diameter, and the increase in the particles size [63].

### In vitro cytotoxicity assay

#### Effect of different concentrations of 6MP, 6MP-CNPs complexes, and 6MP-CNPs-AuNPs nanocomposites on cellular proliferation of MCF7 cell line

In the case of free 6MP, the maximum inhibition was 39% at 100 μM. 6MP was designed to interfere with nucleic acid biosynthesis and in order to exert its cytotoxic effect, it requires activation. However, it can be converted into anticancer-active intermediates via enzymatic reaction [64]. Improvement of the cytotoxicity of 6MP-CNPs and 6MP-CNPs-AuNPs nanocomposites was probably due to the better accumulation of the drug at its site of action by targeted delivery via passive EPR (Fig. 1). Generally, nanocarriers are non-specifically internalized into cells via endocytosis or phagocytosis compared to the passive diffusion mechanism of free drugs into the cell [65]. Positively charged nanocarriers like 6MP-CNPs and 6MP-CNPs-AuNPs were known to facilitate their penetration through the negatively charged cancer cell membrane [66]. So, the electrostatic interaction could enhance the absorption of our proposed nanocarriers by MCF7cells. 6MP-CNPs-AuNPs nanocomposites exhibited slightly more inhibition than 6MP-CNPs complexes probably due to higher surface area which might lead to more drug loading and relatively higher zeta potential which lead to better accumulation at the tumor side. Our results were in accordance with previous work with in vivo study Govindappa et al. [67], they showed that the chitosan improved the lethal dose (LD50 cut off) of 6-MP-CNPs (1000 mg/kg b.w) against 6-MP (500 mg/kg b.w) and also significantly (p ≤ 0.05) reduces the toxic adverse effect (28-day repeated oral dose) on hemato-biochemical and hepato-renal histological profiles. Acute and subacute toxicity of 6MP-CNPs (vs 6-MP) shows reduced lethality (LD50) and has adverse effects on blood, liver, and renal profiles. Overall, the findings of this study suggest the importance of 6MP-CNPs as a drug-delivery system to reduce toxic profile and to improve therapeutic dosage in the in vivo preclinical studies.

#### Chemo-photothermal combined treatment

After laser exposure, the enhancement of 6MP cytotoxicity was probably due to the highlighting loss of cell membrane integrity, which is severely affected by heat shock. This probably enhances permeability and lead to the accumulation of drug on the tumor side [68]. Laser irradiation of 6MP-CNPs-AuNPs produced a relatively higher reduction in cell viability compared to 6MP-CNPs (Fig. 8). The reason is attributed to the photothermal effect of AuNPs with diameters ranging from 10 to 30 nm, which are ideal for biomedical applications [69, 70]. As such, AuNPs are efficient agents to induce precise heating, leading to less damage to surrounding tissues while affecting the more thermos-sensitive malignant cells [71]. By using localized hyperthermia combined with chemotherapy delivery, a reasonable reduction in cell viability was achieved, which overcome the need for higher concentrations of native 6MP for equivalent effect. With the help of our proposed nanocarriers, reduction of the effective concentration of 6MP resulted in reducing its side effects and increasing its therapeutic efficiency.

## Conclusion

The present work introduced a method for fast, highly stable, and low-cost synthesis of chitosan nanoparticles (CNPs) based on the ionic gelation method. The mean particle size of CNPs ranged from 19 to 140 nm with zeta potential ranging from 10.7 to 36.9 mV respectively with different ratios between chitosan and TPP. CNPs encapsulated 6-Mercaptopurine were prepared using the modified ionic gelation method with subsequently loading on AuNPs to form novel 6MP-CNPs-AuNPs nanocomposites. The as-prepared 6MP-CNPs-AuNPs nanocomposite showed high stability with a zeta potential of 44.4 mV. Both 6MP-CNPs and 6MP-CNPs-AuNPs were characterized by their intracellular drug delivery. The effect of native 6MP, 6MP-CNPs, and 6MP-CNPs-AuNPs on the MCF7 cell line was examined. Free 6MP produces maximum inhibition of 39% at 100 μM moreover, 6MP-CNPs and 6MP-CNPs-AuNPs produce a significant decrease in cell viability reaching IC_50_ at 9.3 and 8.7 μM respectively. The results showed that the encapsulation of 6MP in CNPs significantly enhanced the anti-proliferation activity of 6MP, which can be the result of enhancing the intracellular uptake of 6MP by the endocytosis mechanism. Therefore, the improved anti-proliferation activity of 6MP by CNPs could reduce the overall concentration of the drug and decrease its side effects during cancer treatment. Moreover, as-prepared 6MP-CNPs-AuNPs nanocomposite was utilized as a delivery photothermal combined therapeutic agent. Laser irradiation of 6MP-CNP-AuNPs nanocomposite resulted in the highest inhibition in cell viability and reached IC_50_ of 4.4 µM. A combination of therapeutic approaches against cancer cells was applied to improve the bioavailability, prevent the appearance of resistance to chemotherapy and reduce the dosage of chemotherapeutic agents. Future work is in progress to investigate the positive effects of 6MP-CNPs-AuNPs nanocomposite in vivo.

## Data Availability

The datasets used and/or analyzed during the current study are available from the corresponding author on reasonable request.
